# Decease of Prof. Harrison

**Published:** 1849-09

**Authors:** 


*Decease of Prof. Harrison.*—We are pained to see announced the decease of Prof. Harrison, of Cincinnati. Prof. H. was connected with the Ohio Medical College, and was, also, one of the Editors of the Western Lancet. He was a zealous and successful teacher, an able writer, and a judicious, skillful practitioner. In his decease, the College with which he was connected, his brethren and the community of Cincinnati, and the medical profession, have lost a valued, useful, and distinguished member. Prof. H. took an active and prominent part in the American Medical Association, and was elected first vice president at the meeting at Boston, Mass. in May last.

